# Biodegradability of Polyolefin-Based Compositions: Effect of Natural Rubber

**DOI:** 10.3390/polym14030530

**Published:** 2022-01-28

**Authors:** Ivetta Varyan, Natalya Kolesnikova, Huaizhong Xu, Polina Tyubaeva, Anatoly Popov

**Affiliations:** 1 Academic Department of Innovational Materials and Technologies Chemistry, Plekhanov Russian University of Economics, 117997 Moscow, Russia; anatoly.popov@mail.ru; 2 Department of Biological and Chemical Physics of Polymers, Emanuel Institute of Biochemical Physics, Russian Academy of Sciences, 119334 Moscow, Russia; kolesnikova@sky.chph.ras.ru; 3 Department of Biobased Materials Science, Kyoto Institute of Technology, Kyoto 606-8585, Japan; xhz2008@kit.ac.jp

**Keywords:** biodegradation, life cycle, polyethylene, natural rubber

## Abstract

Recently, environmental problems caused by the overproduction and consumption of synthetic polymer materials led to an urgent need to develop efficient methods for processing plastics. The accumulation of polymer waste for their subsequent incineration does not solve the problem due to the limited areas of landfills for waste storage. In addition, the incineration of polymer waste can cause toxic air pollution, which, in turn, does not contribute to an improvement in the environmental situation. Recycling plastics, although a more environmentally friendly waste disposal method, requires significant labor and energy costs and can be performed a limited number of times. Thus, the most promising solution to this problem is the creation of biodegradable polymers capable of degradation with the formation of simpler chemical structures (water, carbon dioxide, biomass, etc.), which are easily included in the metabolic processes of natural biological systems. The article provides an overview of the main trends in the creation of biodegradable composites for the needs of agriculture. Also, the article proposes a new composition based on polyethylene with natural rubber that surpasses existing biodegradable materials in a number of physical and mechanical characteristics and has the ability to complete biodegradation in 60 months. It is shown that the studies carried out to date indicate that these composites are highly promising for the creation of biodegradable packaging materials with good performance characteristics. Thus, it was concluded that further research on composites based on polyethylene and natural rubber is important.

## 1. Introduction

Synthetic polymers are actively used in science, technology, agriculture, construction, medicine, as well as in almost all spheres of everyday life [1,2,3,4,5,6,7,8,9,10]. Due to their elasticity, durability, and high resistance to mechanical, chemical, and biological influences, synthetic polymers are used in the production of films and fibers used as packaging materials, containers, electrical and heat insulating materials, and much more. At the same time, about half of the production volume (more than 178 million tons per year) and consumption of synthetic polymers in the world falls on polyolefins [8,10], in particular, polyethylene (PE). According to studies [11], in the near future, the trend towards an increase in the production of synthetic polymers will continue.

**Figure 1:**
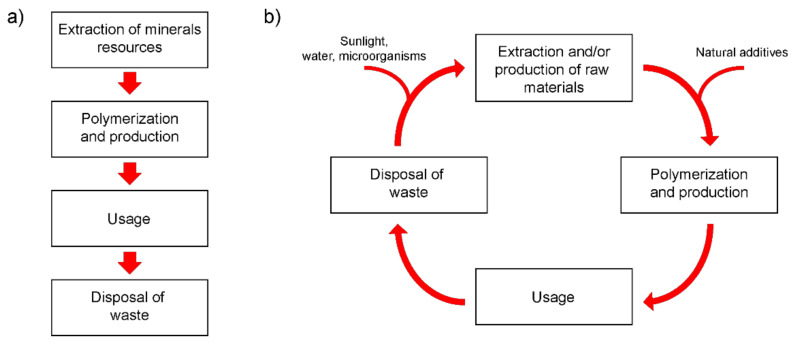
(**a**) Stages of life cycle of polymeric materials. Cycle begins with extraction of fossil resources and ends with accumulation and, possibly, partial processing of polymer waste. (**b**) Expected life cycle of biodegradable polymer materials. At end of their service life, polymers decompose into simple chemical structures due to vital activity of bacteria. Further, decomposition products can be used to produce new materials.

At the same time, large scale production and consumption of polyolefins is one of the dominant factors responsible for the accumulation of plastic waste in the environment [7,10]. This fact is clearly visible in Figure 1a depicting typical lifecycle stages of nonbiodegradable polymeric materials. Ironically, it is the high resistance of polyolefins to external influences that made them such a commercially successful polymer for the production of packaging materials, which is the main disadvantage of this material at the end of its life and ending up in waste. The negative impact of environmentally resistant wastes containing significant amounts of polyolefins on the environment is becoming increasingly threatening. At the same time, the methods of storage and disposal of plastic waste are limited. For example, the incineration of used plastic packaging materials can cause toxic air pollution, and landfills for this type of waste collection and disposal are limited. Recycling of plastic waste is a rather expensive process and is currently carried out only for special types of plastics in relatively small quantities. In addition, this process can release significant amounts of toxic chemicals (e.g., ethylene oxide, benzene, xylene) into the air and water, which can cause serious health problems in humans, including cancer, birth defects, and damage to the nervous system. Attempts to create fully biodegradable polymers with attractive manufacturing costs for commercial use and acceptable performance to replace polyolefins were so far unsuccessful. Therefore, it is becoming increasingly obvious that the best solution to the problem of environmental pollution with plastic waste is the development of technologies for converting existing and commercially used plastics into biodegradable [7,12]. Thus, the modification of a synthetic polymer matrix by introducing additives that initiate the rapid degradation of the polymer makes it possible to obtain new composite materials with increased biodegradability at the end of their life cycle, see Figure 1b.

At the same time, it is expected that the commercial profitability of the production of such composite materials will be quite high due to the absence of expensive synthesis steps in the production cycle [12]. In this case, the presence of a synthetic polymer in the composition of the composite determines the required operational and technological properties, as well as the possibility of secondary use. In turn, the type and concentration of the additive introduced into the polymer determines the rate of its biodegradation.

This article provides an overview of recent advances in the production and study of the properties of biodegradable polymer composites based on polyolefins. The use of polyolefins, especially PE, as a polymer matrix for such composites is due to the importance of their use and a large amount of waste based on them. At the same time, special attention is paid to composites produced using natural fillers such as, for example, starch and natural rubber. It is shown that the progress achieved to date in the production and study of the properties of polymer composites indicates the prospects for the creation of biodegradable materials with operational properties that allow them to be used as packaging materials for the needs of agriculture in the near future. Additional laboratory research is also required before the introduction of these polymer composites into industrial production.

## 2. The Current State of the Problem of Processing Polymer Waste

Polyolefins are polymers made from simple olefins (alkenes) with the general formula C_n_H_2n_ acting as monomers. For example, polyethylene is a polyolefin obtained by the polymerization of olefinic ethylene. Due to its relatively low price and high-performance characteristics, PE is currently the most produced polymer material. So, according to [1,2,3], more than half of the total volume of plastics production in the world falls on polyolefins, in particular PE. At the same time, the accumulation of such large volumes of plastics in the environment and their impact on the environment is becoming more and more threatening. According to [2], in Canada alone, 87% of plastic waste (about 9.7 million tons of plastic mass per year) ends up in landfill, not recycling. According to other data [2], in the United States from 1990 to 2017, an average of 20.8 million tons of plastic waste was produced and collected in landfills. As the analysis shows, the bulk of this waste is various packaging materials based on PE. At the same time, up to 40% of the volume of plastic waste can fall on disposable packaging. Studies [1,2,3,4,5,6,7,8] showed that around 6.5% of the global packaging waste collected is in European countries. Another 0.33% is accounted for by Australia.

The threat of environmental disaster, as well as the depletion of resources due to the large-scale production of synthetic polymers, pushed researchers and manufacturers around the world to explore the possibility of recycling and reusing plastic waste as raw materials for the production of new products. However, a number of related technical problems (such as the discrepancy between the rates and volumes of accumulation of polymer waste of a certain type with the demand for their consumption), the need for significant labor and energy costs, as well as the limited number of recycling cycles make it impossible to solve the problem of recycling plastic waste only by reusing recycled polymer materials. Therefore, as an additional measure to combat the accumulation of plastic waste, the creation of biodegradable composite materials capable of decomposing into simple chemical compounds (water, carbon dioxide, biomass, etc.) under natural conditions as a result of the vital activity of common types of microorganisms is currently being considered.

## 3. Industry of Biodegradable Polymer Materials

**Figure 2:**
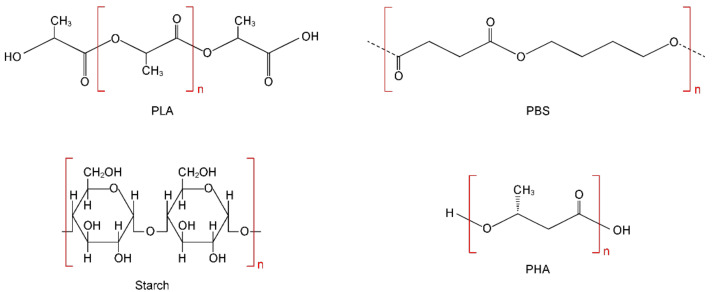
Structural formulas of some biodegradable polymers.

As of 2019, the global production capacity for bioplastics produced from renewable biomass sources has reached 2.11 million tons. Of these, approximately 1.17 million tons (55.5%) are various biodegradable polymeric materials [7,8,9,10]. There are currently over 20 groups of biodegradable polymers. However, only 4 out of 20 of these groups are commercially produced: (i) polylactic acid (PLA); (ii) starch-based plastics; (iii) polybutylene based polymers (PBS/PBAT); and (iv) polyhydroxyalkanoates (PHA). It is these four groups that account for up to 95% of all production capacity for the production of biodegradable plastics in the world. In view of the extreme practical importance of these bioplastics, let us dwell in more detail on the characteristics of each of them. The structural formulas of these bioplastics are presented in Figure 2.

PLA is relatively inexpensive and has a number of attractive mechanical properties that make it a very popular material. As of 2019, the volume of PLA produced was about 290 thousand tons [13]. PLA production relies heavily on plant materials such as cassava, potatoes, corn, and sugarcane [7]. Despite attempts to use other sources of raw materials, such as, for example, agricultural waste, cellulosic materials, or greenhouse gases (carbon dioxide and methane), these technologies are still under development [13].

Starch-based plastics are relatively inexpensive to manufacture, making them a very popular biodegradable material. Thus, the total production of starch-based plastics in 2019 was about 450,000 tons [13,14,15,16]. Natural starch consists of two types of glucose polymers, namely 10–20% amylose (inner part) and 80–90% amylopectin (shell). Both polymers are composed of α-glucose monomers and have the composition ${\left(C_{6}H_{10}O_{5}\right)}_{n}$. Starch is a biodegradable polymer that can be easily processed to form thin film products with low oxygen permeability. However, pure starch has poor water resistance and mechanical strength. Therefore, starch is often blended with other polymers to achieve the desired mechanical properties in the commercial plastics industry. Thus, starch-based plastics are mixtures with plastics such as polylactic acid, polybutylene succinate, polybutylene adipate terephthalate, and others [7]. Another starch-based material is thermoplastic starch (TPS), which is obtained from natural starch by heating and adding various types of plasticizers.

PBS/PBAT are fossil-based biodegradable polymers. The current production capacity of PBS/PBAT polymers is 370 thousand tons [13,16,17,18,19,20,21]. There are two main pathways for the synthesis of PBS: the transesterification process (from succinate diesters) and the direct esterification process starting with diacid. Since PBS is naturally degraded to water and CO_2_, it can be used as a biodegradable alternative to some common plastics.

Polyhydroxyalkanoates (PHAs) are another important group of biodegradable polymers. Due to the high cost of production, the current production capacity of PHA is only 25,000 tons [13,22,23,24,25]. However, it is expected that in the coming years, the production of PHA in European countries will increase significantly [7,13]. PHA are produced by microbial fermentation, mainly using sugar or oil. PHA is nontoxic and has good UV resistance, as well as satisfactory physical and chemical properties. The use of PHA is still very limited due to its poor mechanical properties, incompatibility with traditional heat treatment methods, and its tendency toward thermal decomposition.

In addition to the four main biodegradable polymers mentioned above, there are many other types of biodegradable plastics, including water soluble PVOH (polyvinyl alcohol), PPC (propylene carbonate), PCL (polycaprolactone) and others. However, these materials need further research and laboratory tests before they can be considered for commercial use.

## 4. Factors Contributing to the Biodegradation of Polymers

The biodegradation process can be defined as the process of changing the chemical structure of a polymer from a more complex to a simpler one under the influence of various biological factors, such as soil bacteria, mold fungi, and various atmospheric microorganisms. In addition, various physical (ultraviolet radiation, temperature, humidity) and chemical (presence of certain reagents in aqueous media) phenomena can be attributed to the number of factors affecting the biodegradation process. All these factors contribute to the destruction of polymer molecules with the formation of simpler chemical structures that are easily included in the metabolic processes of natural biological systems.

The role of microorganisms in the biodegradation of polymers is very important since different types of microorganisms destroy only certain groups of polymers. So, for example, of the whole variety of available microorganisms, only 17 genera of bacteria and 9 genera of molds have the ability to destroy PE [2,10,26,27]. The growth rate of a population of microorganisms is significantly influenced by several external factors, including the presence or absence of water, temperature, redox potential, and others. At the same time, the polymeric materials themselves are a nutrient source of organic matter for microorganisms. Consequently, the ability of polymers to degrade by microorganisms largely depends on the structural characteristics of the polymers themselves.

In this case, the most important characteristics that determine the degradability of polymers include the chemical nature of the polymer, branching and flexibility of macrochains, the presence and nature of side groups, molecular weight, and some others. Let us consider some of these characteristics of polymers in more detail:

1. Molecular weight. Molecular weight plays a critical role in determining many of the properties of a polymer. In particular, the molecular weight of polymers has a significant effect on its biodegradability. For example, the biodegradability of polymers decreases with increasing molecular weight.
2. Shape and size. The shape and size of the polymer also play an important role in the biodegradation process. Polymers with a larger surface area can degrade much faster than polymers with a small surface area.
3. Additives. Any nonpolymeric additives, be it colorants, fillers, wastes, or residues of catalysts used for polymerization, affect the degradability of plastics. From a practical point of view, biodegradable additives are of particular interest, which accelerate the degradation of polymers, allowing microorganisms to use the carbon in the polymer chain as an energy source. Thus, biodegradable additives can convert degradation of plastic to biodegradation. In this case, instead of being degraded by environmental factors (such as sunlight or heat), biodegradable additives allow polymers to be degraded by microorganisms and bacteria. Typically, biodegradable additives accelerate the rate of degradation by reducing the strength of certain polymer properties and increasing their attractiveness to microorganisms.
4. Biosurfactants. Biosurfactants are microbial surface-active compounds. Due to their low toxicity and high biodegradability, biosurfactants, when added to polymers, can enhance the biodegradability of the latter. In addition, due to the presence of certain functional groups, biosurfactants make it possible to observe biological activity even under conditions of extreme temperatures, pH and salinity.

**Figure 3:**
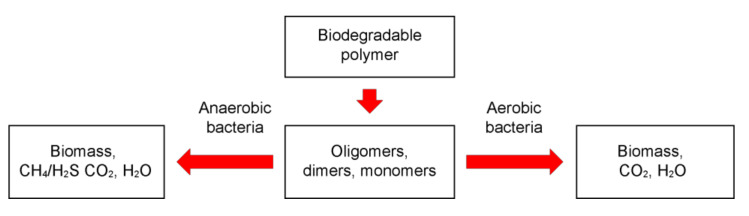
Biodegradation of polymeric materials under aerobic and anaerobic conditions.

Moreover, both the biodegradation mechanism and the group of microorganisms responsible for it are often determined not by the characteristics of the plastic, but by the environmental conditions. So, in anoxic conditions, the activity of anaerobic microorganisms leads to the decomposition of synthetic polymers with the formation of microbial biomass, CO_2_, CH_4_ and H_2_O (under the action of methanogenic bacteria) or H_2_S, CO_2,_ and H_2_O (under the action of sulfidogenic bacteria) as the main products of biodegradation, see Figure 3. In turn, in the presence of oxygen, aerobic microorganisms are mainly responsible for the biodegradation of the polymer material with the formation of microbial biomass, CO_2_ and H_2_O. Next, we will consider in more detail the environmental factors and the mechanisms of their influence on the process of biodegradation of polymers.

1. Humidity. The growth and reproduction of microbes requires significant amounts of water. In addition, high humidity stimulates the hydrolysis process by increasing the number of chain-breaking reactions. Consequently, the rate of polymer degradation increases in the presence of sufficient moisture.
2. pH and temperature. pH can change the rate of hydrolysis reactions. For example, for PLA, the optimal rate of hydrolysis is observed at pH = 5. The degradation products of polymers can also affect the pH value, and therefore affect the rate of decomposition and growth of microbes. Likewise, the softening temperature of the polymer significantly affects the enzymatic degradation. Polymers with a higher melting point are less biodegradable.
3. Enzyme characteristics. Enzymes have unique active sites and are capable of biodegradation of various types of polymers. For example, straight chain polyesters derived from dibasic monomers with 6 to 12 carbon atoms are rapidly degraded by enzymes produced by the fungi *A. flavus* and *A. niger*.

## 5. Mechanisms of Biodegradation of Plastic by Microbes

As noted above, microbes (bacteria or fungi) are capable of producing extracellular enzymes that aid in the degradation of various types of plastics. In this case, polymers decompose to CO_2_ and H_2_O through various metabolic and enzymatic mechanisms [2]. The nature and catalytic activity of enzymes varies with the species of microbes. For example, *Bacillus spp*. and *Brevibacillus spp*. produce proteases involved in the degradation of various polymers [28,29,30,31]. Molds often contain laccase, which catalyzes the reactions of aromatic and nonaromatic compounds during oxidation. These microbial enzymes also affect the rate of biodegradation of polymers. The primary process in plastic biodegradation is the adhesion of microbes to the polymer surface, followed by colonization. Enzymatic hydrolysis of plastics includes two stages: (1) attachment of the enzyme to the polymer surface, and then, (2) hydrolytic fission. Decomposition products of polymers (oligomers, dimers, and monomers) have a very low molecular weight and are ultimately converted to CO_2_ and H_2_O as a result of mineralization [2]. Under aerobic conditions, oxygen is used by bacteria as an electron acceptor, followed by the synthesis of smaller organic compounds, and thus CO_2_ and H_2_O are produced as end products. Under anaerobic conditions, polymers are destroyed by microorganisms in the absence of oxygen. At the same time, sulfates, nitrates, iron, carbon dioxide, and manganese are used as electron acceptors by anaerobic bacteria [32].

## 6. Polymer Composites with Natural Additives

The most promising method for creating biodegradable polymers is the modification of the polymer matrix by introducing into the structure of the main chain of additives that are sensitive to the action of destructive agents. For example, oxo-degradable additives based on transition metal salts of cobalt, nickel, or iron are widely used to impart biodegradability to polyolefins [2,7,10]. Under natural conditions, the decomposition of such materials occurs in two stages. The first stage is the decomposition of the plastic product into fragments, induced by the action of sunlight and oxygen. At the second stage, there is a complete or partial decomposition of plastic fragments due to the vital activity of microorganisms. Note that when disposing of waste in real conditions, the simultaneous presence of all factors necessary for the implementation of the first stage of plastic decomposition is difficult to achieve. Therefore, as studies show [2,7], over a period of 350 days, only about 15% of the oxo-decomposable PE placed in the soil decomposes to carbon dioxide.

An alternative, which recently become widespread, is the creation of composite materials based on a mixture of a polymer with natural or synthetic biodegradable additives [33,34,35,36,37,38,39]. The undoubted advantage of such materials is the controlled resistance to the action of microorganisms, which makes it possible to obtain compositions both more resistant to biological influences and, conversely, easily biodegradable. An example of such polymer compositions are graft copolymers of starch and methyl acrylate, the films of which are used for mulching soil in agriculture.

Also known are a number of polyolefin composites with the addition of natural biodegradable polymers, such as polyhydroxyalkanoates or polylactic acid, requiring specific composting conditions. For example, the biodegradability of polylactic acid is fully realized only at elevated temperatures (50–60 °C). In turn, materials based on polyhydroxyalkanoates are highly biodegradable and biocompatible. However, the mechanical and physical properties of such materials are rather poor, which limits the scope of their practical application. In addition, the technological difficulties in obtaining polyesters by biosynthesis determine the high cost of materials based on them. To improve mechanical properties, biopolymer mixtures were developed, transformed into micro- or nanofibrillar biocomposite materials, in which a biopolymer with a higher melting point acts as a reinforcing element and a biopolymer with a lower melting point acts as a matrix. For example, there are studies [10] devoted to composites based on polylactic acid obtained by microinjection, containing polybutylene succinate nanofibrils 10–40 wt.%, 3–10 wt.% polybutylene adipate terephthalate 20 wt.% polycaprolactone.

**Figure 4:**
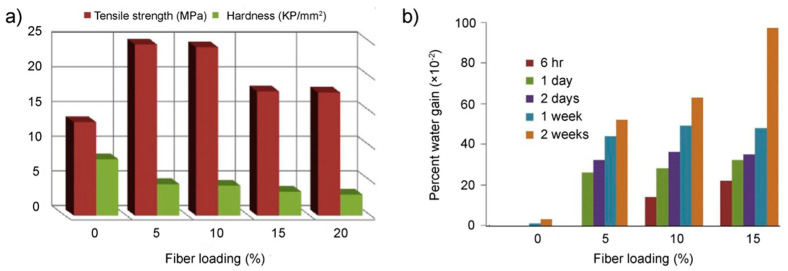
(**a**) Tensile strength and hardness; (**b**) percentage of water absorption of PE/corn husk fiber composites with different fiber percentages. Adapted from Ahmed Youssef et al. [40].

**Figure 5:**
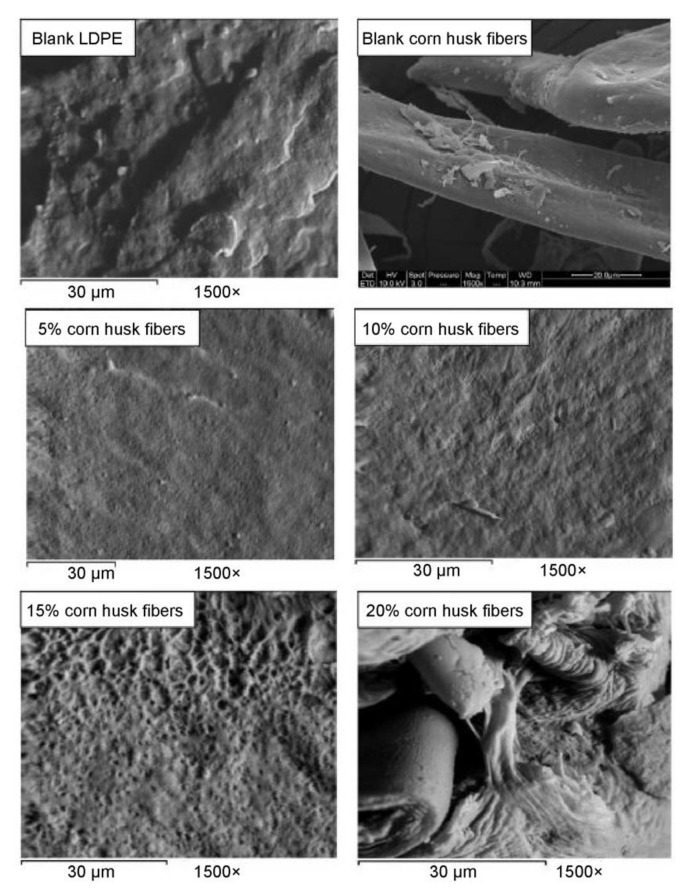
Scanning electron microscopy (SEM) micrographs of composites based on PE and corn husk fibers. Content of corn husk fibers in composites varies from 0–20%. Adapted from Ahmed Youssef et al. [40].

The addition of natural fillers to a synthetic polymer matrix can significantly affect not only biodegradability, but also other properties of materials. In this case, it is possible to obtain polymers with improved mechanical or thermal properties. At the same time, the use of natural fillers obtained from the waste of agricultural production can significantly reduce the cost of such materials, which makes their profitability very high. For example, in Ref. [40], composite materials based on low density PE, filled with fibers obtained from corn husks, were obtained and investigated. In particular, the influence of fiber components on the mechanical, thermal properties, water absorption, and crystalline properties of reinforced PE/corn husk fiber composites was studied. Corn husk fibers (surrounding the corncob) for research were obtained from a farmers’ market (El Menufia province, Egypt). The corn husks were dried, crushed, and sieved so that the particle size was no more than 125 microns. Chemical analysis of corn husks revealed the following fiber composition: cellulose 43%, hemicellulose 31%, lignin 22%, and ash 1.9%. Before preparing the composites, all fibers were dried at 80 °C until the moisture content was reduced to a level of 1–2%. Low density PE was blended in a mixer (Haake Rheomex TW100, intermeshing twin screw extruder, Thermo scientific, Waltham, MA, USA) at 160 °C and a rotor speed of about 60 rpm. The prepared mixture was removed from the mixing chamber, cooled, and cut into small pieces suitable for feeding into a press. The samples prepared in this way were plates with a thickness of 3–4 mm. After the thermoplastic matrix was melted, corn husk powder was added and mixed. Compression molding of the samples was carried out at a temperature of 175 °C and a pressure of 5 MPa for 5 min. Then each sample was cooled under pressure to room temperature. The study of the properties of composites obtained in this way showed that the mechanical properties (modulus and tensile strength) were significantly improved due to the presence of corn husk in the fibers, see Figure 4a. In this case, the hardness of the composites decreased with an increase in the fiber content. As seen in Figure 4b, the water absorption of the composite samples increased due to the presence of corn husk fibers. In addition, the thermal stability of the prepared composites was significantly improved in comparison with the samples of the original PE. Micrographs of composites based on PE and corn husk fibers are shown in Figure 5. The micrographs clearly show that the degree of loading of the polymer matrix with corn husk fibers has a great influence on their internal structure. Thus, the surfaces of the PE/corn husk fiber composites (fiber content 5%, 10% and 15%) show a smooth topography. This indicates a high degree of compatibility between the polymer matrix and corn husk fibers and, as a consequence, the appearance of a composite with modified properties. At that time, a polymer loading of 20% fiber or more leads to the emergence of a highly inhomogeneous structure, which casts doubt on the ability of the fibers to modify the polymer matrix at such significant loading. Thus, the analysis of the photomicrographs confirms the tensile strength measurements shown in Figure 4a.

Another example of modification of the properties of PE is the work [41]. Here, to improve the performance properties of PE, a natural filler based on rice husk ash was used. For this study, high density PE manufactured by Reliance Industries Limited (India) was used. Rice husks were collected in rice mills, washed with distilled water to remove sand and other contaminants, and dried in an oven at 100 °C. Further, the husk was fired in a muffle furnace for 6 h at various temperatures in the range of 500–700 °C. Composite samples prepared both with the use of a compatibilizer (the product of the reaction between maleic anhydride and PE in the presence of dicumyl peroxide) and without it were studied. For samples without compatibilizer, only amorphous ash obtained by roasting rice husks at 550 °C was used. To prepare composites using the HDPE compatibilizer, the compatibilizer and rice husk ash were mixed at 145 °C in a Haake Polylab (Thermo scientific, Waltham, MA, USA system equipped with a roller rotor. Before mixing, the rice husk ash was heated in an air oven to remove moisture. During stirring, various amounts of ash were used, ranging from 0 to 2.5 wt.%. In this case, the amount of compatibilizer remained constant (15% of the weight of the entire mixture). Composites obtained without using a compatibilizer exhibited lower mechanical properties than the original polymer. In contrast, composites prepared using the compatibilizer had significantly better mechanical properties. In particular, it was possible to achieve an improvement in indicators such as tensile strength, modulus of elasticity and elongation. It was also found that composites with improved properties have a more uniform structure. The best mechanical properties were observed at an ash and compatibilizer content of 1.5% and 15%, respectively. Thus, the possibility of using rice husk ash as a reinforcing filler in the processing of high-density PE was shown.

We also note that studies of the possibility of changing the properties of PE by introducing natural fillers based on cellulose materials were carried out in other works using wood flour, flax seeds, sisal, and hemp, as well as banana flour and other fillers [42,43,44,45,46]. The composition and main features of some biodegradable polymer composites with natural fillers are summarized in Table 1.

## 7. Composites Based on Polyethylene and Soy Protein

Research on the creation of biodegradable polymers using natural fillers capable of significantly accelerating plastic degradation is of particular interest for this review. One example of this kind of research is the work on the use of soy protein as an additive that accelerates the biodegradability of PE, published in Ref. [47]. In this work, commercial low-density PE manufactured by Thukral Trading Co. (Ludhiana, India) was used. PE in the form of granules was dissolved in paraxylene and precipitated by the addition of methanol. Then, PE was irradiated in a cobalt-60 source (BARC, Mumbai, India) at a constant dose rate of 3.40 kGy/h. Soy protein was grafted onto preirradiated PE with benzoyl peroxide as a radical initiator. The maximum percentage of inoculation (135%) was obtained at a benzoyl peroxide concentration of 2.15 × 102 mol/L and a temperature of 70 °C for 150 min with 0.2 g of polyethylene, 0.3 g of soy protein and 40 mL of water.

**Figure 6:**
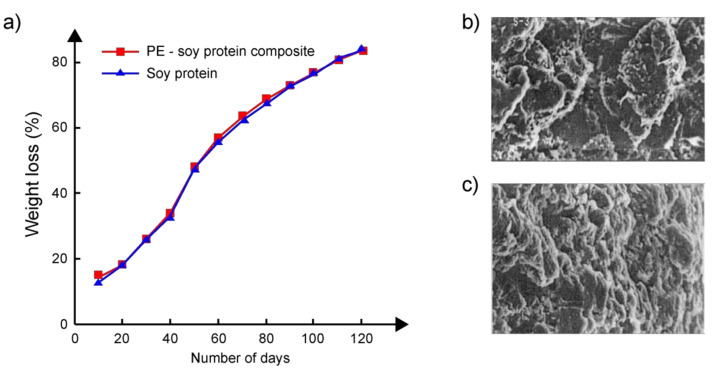
(**a**) Percentage weight loss of soy protein and PE/soy protein composite as a function of residence time in soil. SEM micrographs of a sample of the PE/soy protein composite (**b**) before and (**c**) after biodegradation in soil (magnification 2000×). Adapted from Inderjeet Kaur et al. [47].

To study the biodegradability of soy protein (SP) grafted polyethylene, hereinafter referred to as the PE/SP composite, the weight loss of samples placed in soil was measured. For this, 1200 g of garden soil was placed in special containers. A weighed amount (1 g) of each of the samples (pure PE and PE/SP composite) wrapped in a synthetic mesh was placed separately in each container so that the samples were completely covered with soil. The pots were covered with aluminum foil and kept at room temperature. Sample weights were measured every 10 days. The results of these measurements are shown in Figure 6a. The percentage of weight loss due to degradation was determined as the difference between the weight of the sample taken on a particular day (i.e., every 10 days) and the initial weight. The figure shows that the percentage weight loss of both samples increased continuously over four months and eventually reached 76% and 74% for the PE/SP composite and pure SP samples, respectively. Analysis of micrographs of samples of PE/SP composites obtained using a scanning electron microscope (Figure 6b,c) also showed noticeable changes in the structure of the surface of the samples as a result of their stay in the soil. As the authors point out, the decrease in percentage weight loss over time is associated with the penetration of microorganisms into the samples, followed by moisture absorption. At the same time, for the initial PE placed in similar conditions, weight loss in all measurements was 0% even after four months of observation. On the contrary, pure SP placed in the same soil completely decomposed within 10–20 days. Analysis of the soil showed that the soil without the sample, as well as the soil containing the pure PE sample, showed only a slight increase in the number of bacteria during the study.

During the same time, an abundant growth of bacterial colonies was observed in the soil with the samples of PE/SP composites. Also, comparing the increase and decrease in the number of bacterial colonies with the percentage weight loss of polymer samples in the soil, it was found that the increase and decrease in the percentage weight loss occurred in parallel with the growth and decrease in the number of colonies. These observations indicate microbial activity as the dominant cause of polymer weight loss over time.

## 8. Composites Based on Polyethylene and Natural Rubber

Despite the significant increase in the biodegradability of polymer composites discussed in the previous section, the mechanical properties of such materials are still inferior to traditional plastics, which limits their practical application. This fact stimulates the search for new materials for creating polymer composites with characteristics acceptable for their commercial use. As studies show [12,34,48,49,50,51,52,53,54,55] natural rubber (NR) additives have great potential for use as a component for initiating biodegradation processes in composites based on PE and other polymers [55,56,57,58,59]. NR-doped composites can be quite susceptible to biodegradation by a wide variety of microorganisms, including a wide variety of bacteria and molds [60,61,62,63,64,65,66,67,68,69,70,71]. According to the data in [52], when creating composites based on PE with the addition of NR, the main component of natural rubber, polyisoprene, forms flexible nano- or micro-sized droplets in a polyethylene matrix. The droplet size depends on the content and technology of mixing NR and PE. Due to the uniform distribution of droplets in the polymer matrix, the mechanical properties of PE/NR composites can remain at a level acceptable for their commercial use as packaging materials even with large loads of the polymer matrix. This forces us to turn to the study of the properties of such composites in more detail.

Studies of the characteristics of biodegradable polymer composites based on low density polyethylene with additives of natural rubber of various concentrations were carried out in the works [48,49,50,51,52]. The biodegradation test was carried out by holding thin films of the samples in the soil for up to 90 days. The measurement results were obtained in accordance with the standard ASTM D 5899. At the same time, the soil used in the measurements consisted of sand, garden soil and horse manure, taken in equal amounts. The soil obtained in this way was kept for two months at 20 °C with daily stirring and maintaining moisture at a level 60%. Samples of the polymer composite were immersed in the prepared soil vertically and kept at 22 °C and 60% relative humidity for 45 and 90 days. To assess the degree of decomposition of the sample material after the test time, changes in appearance, weight and chemical composition were assessed due to the effect of microorganisms in the soil environment. Various mold cultures were used for the tests, including *Trichoderma harzianum Rifai*, *Penicillium chrysogenum Thorn*, *Fiisarhim moniliforme Sheld*, *Chaetommm glohosimi Kunze*, *Trichoderma asperellum Samuels Lieckf and Nireberg,* and others.

As the object of the study, films made of low-density PE grade 15803-020 with the addition of NR (SVR 3L grade, Dong Xoai, Vietnam) or synthetic (NII-3 grade, JSC Sintez Kauchuk, Sterlitamak, Russia) were used. The content of NR in PE films varied at the level of 10–30 wt.%. All the composites used were obtained by mixing PE and NR granules in an argon atmosphere at a temperature of 140 °C, followed by cooling and pressing on a manual hydraulic press to obtain round samples with a diameter of 8 cm and a thickness of 120 μm. A detailed description of the process of making samples is given in the work [52].

The biodegradability of PE/NR composites was assessed during tests in a soil environment. The key results of this study are summarized in Table 2. It was shown that the weight loss of the PE/NR composite is largely dependent on the NR content. Thus, composites with a mass fraction of NR up to 20 wt.% lost less than 2% of their mass after 45 days of being in the soil. Moreover, an increase in the duration of the test to 90 days did not lead to any noticeable increase in this indicator. At the same time, composites with a mass fraction of NR equal to 30 wt.% lost about 2.7% of their mass after 45 days of being in the soil. After 90 days, this figure increased to 7.2%. At the same time, the decrease in the weight of pure NR samples as a result of exposure to the soil environment within 90 days was at the level of 38.3%, which indicates the high biodegradability of natural rubber. Thus, it was found that a higher content of NR leads to more intense changes in the structure of composite materials. This conclusion was confirmed by studies of changes in the appearance, structure, and chemical composition of composites after being in soil, described below.

**Figure 7:**
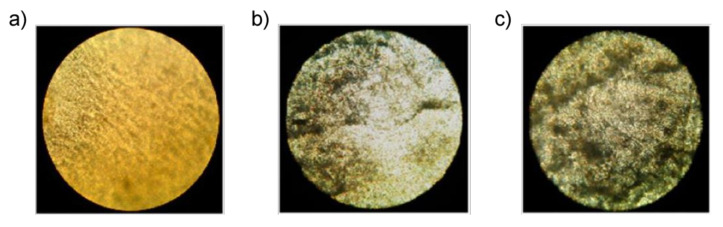
Micrographs of composite PE/NR = 70/30: (**a**) original sample, (**b**) sample after aging in soil for 45 days, (**c**) sample after aging in soil for 90 days. All micrographs were taken in transmitted light at a magnification of 100×. Adapted from Mastalygina Elena et al. [52].

To assess the rate of biodegradation, samples of pure PE and composites of PE/NR were removed from the soil for visual inspection and analysis using optical microscopy. Figure 7 shows photographs of a PE sample in the initial state and after being in the soil environment for 45 and 90 days. As can be seen, the appearance of pure PE samples did not change after exposure to soil. Analysis of micrographs of a sample of pure PE also indicates the absence of any defects, stains, and biological growth after exposure of the image to the soil for 90 days.

In contrast, the PE/NR samples underwent noticeable changes after being in the soil. Numerous defects, darkening of color, and general deterioration of the state of the samples due to the vital activity of soil microorganisms are visible to the naked eye. Also, on the samples, you can observe the loss of transparency and the appearance of colored spots. A more detailed analysis, carried out using optical microscopy, shows that the intensity of biofouling of samples with a content of PE/NR = 70/30 after 90 days in the soil was four points (according to ISO EN 846: 1997). More than 50% of the surface of this sample was covered with germs of microorganisms, which indicates a sufficient content of nutrients in the sample material, promoting the growth of soil microorganisms.

Another criterion for assessing the biodegradability of composite materials based on PE/NR is the study of the rate of water absorption by the samples. Indeed, an increased degree of water absorption promotes the penetration of the metabolic products of microorganisms (acids and enzymes) into polymeric materials, which, in turn, leads to the hydrolysis of NR [12,48,49,50,51,52]. The hydrolysis products have a lower molecular weight and a higher diffusion coefficient through the polymer matrix, and therefore they are able to leave polymer samples, which leads to a loss of material mass. In Ref. [52], to assess the degree of water absorption of polymer films based on a PE/NR composite, as well as pure NR, the samples were placed in distilled water at a temperature of 30 °C. Measurements were carried out according to DIN EN ISO 62: 2008-05. In this case, the changes in the mass of the sample and its appearance after exposure to the simulated environment were evaluated. The measurements were carried out for 45 days until equilibrium water absorption was reached. It was found that NR is characterized by a high degree of water absorption of about 36%, which makes it very vulnerable to soil microorganisms. For synthetic polyisoprene rubber SKI-3, the degree of equilibrium water absorption is lower and is only 18%, which may be due to the difference in the structure of natural and synthetic rubbers. In turn, composites PE/NR have a higher degree of water absorption than pure PE. In addition, the equilibrium water absorption of composites increases with decreasing PE content. Structural changes in PE and NR were observed after exposure to the composites in both aqueous and soil media. It was found that water, including soil moisture, promoted the recrystallization of PE crystals. An increase in the degree of crystallinity of PE can be associated with an increased degree of water absorption and the effect of NR particles on the structure of the material.

As the authors of Ref. [12] point out, the biodegradation of PE/NR composites is a complex process that includes several stages. It is known that bioassimilation and subsequent degradation of polymer materials begins with adhesion and attachment of fungal spores to the polymer surface. After biofouling of the surface, the availability and suitability of the sample material as a nutrient source plays an important role [52]. The convenience of using filled composites for microorganisms mainly depends on the biodegradability of the filler, the diffusion properties of the polymer matrix, the structure of the composite, including the interfacial space, as well as on the degree of water absorption. Under the influence of enzymes of microorganisms and soil moisture on the surface of composite materials, hydrolysis of substances contained in the composite can occur. According to the results of work [12], natural rubber had increased biodegradability (the period of complete degradation of NR is about six months). In addition, the studied PE/NR composites were characterized by an increased degree of water absorption, which facilitates the penetration of vital products of microorganisms (acids and enzymes) into the composite material and leads to hydrolysis and oxidation. In turn, decomposition products, which have a low molecular weight and a higher diffusion coefficient, can leave the samples, which leads to a decrease in the weight of materials when exposed to the soil.

A change in the structure of a polymer material due to the introduction of a filler, as a rule, leads to significant changes in its mechanical properties. For example, the addition of a natural filler to a polymer matrix creates defect zones at the interface between the polymer and filler particles. As a result, the tensile flowability of composites containing filler can be significantly reduced. However, studies of the mechanical properties of PE/NR composites carried out in [48,49,52] unambiguously show that these composites have quite satisfactory elastic properties. This fact is due to the high elasticity of NR and the uniform distribution of NR particles in the polymer matrix.

**Figure 8:**
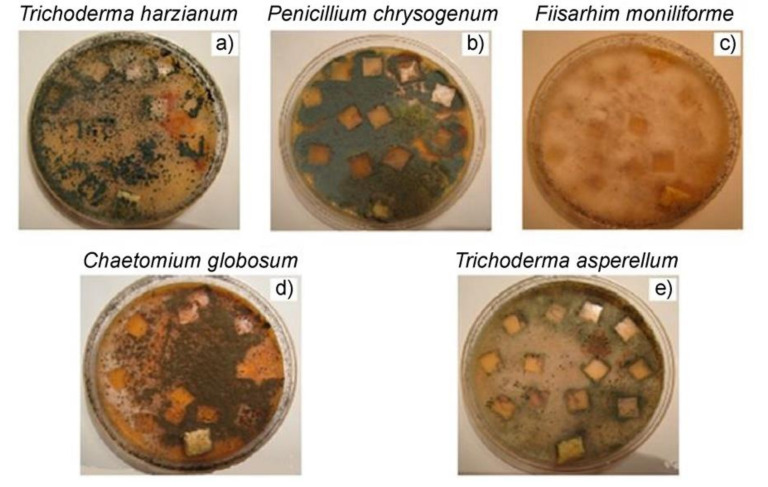
Photographs of composite samples of PE/NR = 70/30 25 days after inoculation with mold fungi (**a**) *Trichoderma harzianum Rifai*, (**b**) *Penicillium chrysogenum Thorn*, (**c**) *Fiisarhim moniliforme Sheld*, (**d**) *Chaetomium globosum Kunze*, (**e**) *Trichoderma asperellum Samuels Lieckf and Nireberg*. Adapted from Mastalygina Elena et al. [52].

Analysis of bioresistance of materials to molds is one of the most common model experiments to determine the biodegradability of polymer composites. It was established in [52] that the fungal cultures *T. harzianum and F. moniliforme* have the most intense effect on the state of PE/NR composites. Thus, these cultures can be called the main biodegradants of PE/NR composites. The colour change of the investigated composite samples was also observed for the cultures of *P. chrysogenum* and *C. globosum*, see Figure 8, which may be associated with metabolites secreted by microorganisms (including pigments). According to the research results, most of the spots of the fungus on the surface of the samples have a general tendency to grow as the content of NR in the composites increases. Apparently, this fact is associated with the greater availability of NR domains in the polymer matrix with a high content of rubber.

Note that the degree of biodegradation of PE/NR composites in the above studies was estimated based on the results of the samples being in the soil for a period of not more than 90 days. At the same time, for an exhaustive analysis of the rate of biodegradation of PE in composite materials of this type, the recommended test duration should be about 1–2 years. In this case, the loss of mass of the samples, a decrease in the degree of crystallinity, as well as the accumulation of oxidation products over time should be used as criteria for assessing the destruction of PE. Thus, it becomes obvious that it is necessary to further study the properties of PE/NR composites.

## 9. Structure and Properties of Composites Based on Polyethylene and Natural Rubber

The main area of commercial application of biodegradable polymeric materials is the creation of various packaging materials used, for example, for the needs of agriculture. Therefore, the most important qualities of such materials, along with their ability to biodegrade in the natural environment, are the presence of physical and chemical properties (elasticity, resistance to mechanical stress, chemical resistance, etc.), which guarantee acceptable performance of materials throughout their life cycle.

**Figure 9:**
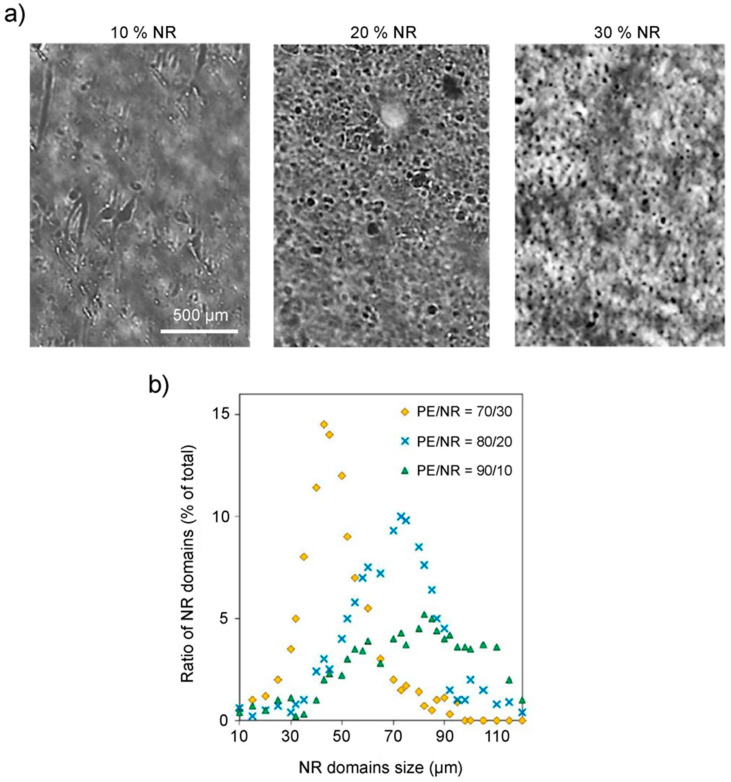
(**a**) Micrographs of composite samples of PE/NR containing 10, 20 and 30 wt.% NR, made in transmitted light at a magnification of 200×. (**b**) Size distribution of NR domains in PE/NR composites containing 10, 20, and 30 wt.% NR. Adapted from Mastalygina Elena et al. [52].

**Figure 10:**
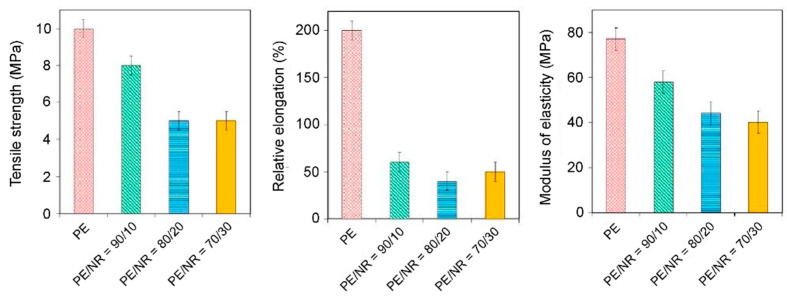
Parameters of ultimate strength at break, elongation at break and modulus of elasticity in tension for pure PE and PE/NR composites with NR content at level of 10, 20, 30 wt.%. Adapted from Mastalygina Elena et al. [52].

The properties of polymeric composite materials based on PE/NR were investigated in [48,49,50,51,52]. Thus, the sizes of the NR domain, as well as the uniformity of their distribution in the PE matrix, were investigated for samples with different NR contents by optical microscopy in transmitted and reflected light at magnifications up to 200×. The microstructure of composite materials based on PE/NR is shown in Figure 9a. The presented micrographs clearly show that the addition of a filler to a polymeric polyethylene matrix leads to significant changes in the morphology of the composite material and in the macromolecular mobility of the boundary layers. The system that appears upon mixing PE with NR is a PE matrix with NR domains distributed inside. The size of rubber domains in composites is on the order of 10–100 μm. At the same time, NR, which has an elastomeric nature, behaves like a flexible dispersed filler, which does not allow considering composite materials based on PE/NR as a mixture of two thermoplastic polymers. As can be seen in Figure 9b, an increase in the NR content in the PE matrix leads to a more uniform distribution of NR domains with a simultaneous decrease in the average domain size. Thus, for composites containing PE and NR in a proportion of 70 to 30 wt.%, the average domain size is about 45 μm. At the same time, for composites containing PE and NR in a proportion of 80 to 20 wt.%, the average domain size is already 75 μm. At a NR content of 10 wt.%, the average domain size is more than 86 μm. In this case, in the samples with small additions of whiskers, an inhomogeneous structure with rather large inclusions of the whiskers phase is observed. Also, in Refs. [48,52], the mechanical properties of polymeric composite materials based on PE/NR were studied. The tests were carried out on a stretching machine in accordance with BS EN ISO 527-1 and BS EN ISO 527-3. In this case, the modulus of elasticity (Young’s modulus) was determined from the stress-strain curves in the region of elastic deformation. It was shown that the microstructure of PE/NR composites determines their tensile behavior. The main parameters of the mechanical properties of the investigated composite films are shown in Figure 10. As can be seen, the addition of NR to PE helps to reduce the relative elongation of PE films at break.

When the NR content is 10 wt.%, the elongation at break decreases four times compared to the elongation of pure PE films. No further changes in the relative elongation at break with an increase in the content of NR in the composite samples were observed. Also, it was found that the tensile strength and Young’s modulus for composite materials on the PE/NR core are approximately two times lower than the values obtained for pure PE. For comparison, we note that when 10–30 wt.% of other dispersed fillers (cellulose, flax straw, wood flour, etc.) are added to PE, the elongation at break decreases by 90%, which clearly indicates the brittle fracture of the material [53,54]. On the other hand, NR domains do not reinforce the polyethylene matrix, unlike most cellulosic fillers. When 10 wt.% NR is added to polyethylene, the tensile strength and elastic modulus are reduced by 20% compared to pure PE. With an increase in the content of NR in the polyethylene matrix to 20 wt.%, a further decrease in strength occurs. However, when the content of NR in the composite is more than 20 wt.%, the tensile strength of the composites ceases to change.

The behavior of PE in PE/NR composites during melting and crystallization was studied by differential scanning calorimetry (DSC) in the temperature range from 40 to 150 °C [52]. DSC analysis was carried out both for the initial samples and for the samples after exposure to aqueous and soil media. According to the DSC results, as the NR content increased, the PE melting peak shifted to the region of lower temperatures. At the same time, the effect of water on the polyethylene component of composites is insignificant. Under the influence of the soil environment for 90 days, the degree of crystallinity of the PE matrix increases from 29% to 34%.

## 10. Conclusions

This article discusses the problem of creating biodegradable polymer composite materials based on polyethylene with the addition of natural fillers. Particular attention is paid to composites based on low density polyethylene with the addition of natural rubber. The relevance of this study is due to the growing threat of an environmental catastrophe in connection with the ever increasing volumes of accumulation of polymer waste in the environment. Based on a review of recent studies in this direction, it is shown that the development of composites based on polyethylene and natural rubber makes it possible to modify the structure and properties of polyethylene so that the rate of its biodegradation in natural conditions significantly increases. The introduction of natural rubber additives into the polymer matrix makes polyethylene more susceptible to decomposition agents such as moisture, aggressive chemicals, oxidants, and metabolic products of soil microorganisms. According to the results of the study of the physical and chemical properties of composites based on polyethylene and natural rubber, their structure and chemical composition, it was found that materials based on polyethylene with natural rubber additives have satisfactory mechanical and technological properties that determine the suitability of such materials for use in agriculture and others. industries as highly biodegradable packaging. Research on composites based on polyethylene with natural rubber filler began only recently. The number of scientific papers devoted to the study of this material and published to date is small. However, due to the combination of a number of positive qualities of this material (low cost and ease of manufacture, good biodegradability and acceptable mechanical properties), we conclude that further research on composites of this type is extremely important.

## Figures and Tables

**Table 1:** Summary of biodegradable polymer composites with natural fillers.

Matrix Material/Filler	Filler Loading (wt.%)	Important Features	Reference
Polyhydroxyalkanoates	0	Highly biodegradable at elevated temperatures. Mechanical properties are rather poor	[3,4,5,6,7,8,9,10]
Polylactic acid	0	Highly biodegradable at elevated temperatures. Mechanical properties are rather poor	[3,4,5,6,7]
Polyethylene/Corn husks	5–20	Low cost, improved mechanical and thermal properties	[40]
Polyethylene/Rice husks	0–2.5	Low cost, improved mechanical properties	[41]
Polyethylene/Soy Protein	Up to 135	Increased biodegradability, environmentally friendly composition	[47]
Polyethylene/Natural rubber	10–30	Low cost and ease of manufacture. Good biodegradability and acceptable mechanical properties	[48,49,50,51,52]

**Table 2:** Change in weight of polyethylene/natural rubber composites after exposure to soil. Adapted from Mastalygina Elena et al. [52].

NR Content in the Composite (wt.%)	Weight Loss after 45 Days in Soil (%)	Weight Loss after 90 Days in Soil (%)
0	0	0
10	1.3	1.3
20	1.5	1.5
30	2.7	7.2
100	16.2	38.3

## Data Availability

Data sharing not applicable.
